# The Price of Success: Immune-Related Adverse Events from Immunotherapy in Lung Cancer

**DOI:** 10.3390/curroncol28060373

**Published:** 2021-11-02

**Authors:** Courtney H. Coschi, Rosalyn A. Juergens

**Affiliations:** 1 Department of Oncology, McMaster University, 699 Concession Street, Hamilton, ON L8V 5C2, Canada; coschic@hhsc.ca; 2 Escarpment Cancer Research Institute, McMaster University, Hamilton, ON L8V 5C2, Canada

**Keywords:** immune checkpoint inhibitor, re-challenge, immunosuppression, immune-related adverse events

## Abstract

Cancer immunotherapy has the goal of enhancing a patient’s intrinsic immune processes in order to mount a successful immune response against tumor cells. Cancer cells actively employ tactics to evade, delay, alter, or attenuate the anti-tumor immune response. Immune checkpoint inhibitors (ICIs) modulate endogenous regulatory immune mechanisms to enhance immune system activation, and have become the mainstay of therapy in many cancer types. This activation occurs broadly and as a result, activation is supraphysiologic and relatively non-specific, which can lead to immune-related adverse events (irAEs), the frequency of which depends on the patient, the cancer type, and the specific ICI antibody. Careful assessment of patients for irAEs through history taking, physical exam, and routine laboratory assessments are key to identifying irAEs at early stages, when they can potentially be managed more easily and before progressing to higher grades or more serious effects. Generally, most patients with low grade irAEs are eligible for re-challenge with ICIs, and the use of corticosteroids to address an irAE is not associated with poorer patient outcomes. This paper reviews immune checkpoint inhibitors (ICIs) including their mechanisms of action, usage, associated irAEs, and their management.

## 1. Introduction

Cancer immunotherapy has the goal of enhancing a patient’s intrinsic immune processes in order to mount a successful immune response against tumor cells. Generally, these approaches fall under a “passive” immunotherapy approach, which uses therapies (e.g., antibody-drug conjugates) to recruit effector cells/molecules of the immune system to directly attack tumor cells, and “active” immune approaches (e.g., CAR-T, type I interferons, anti-CTLA4/PD-1/PD-L1 antibodies), which modulate endogenous regulatory immune mechanisms to enhance immune system activation [1]. Cancer cells actively employ tactics to evade, delay, alter, or attenuate the anti-tumor immune response. Often, strategies that modulate endogenous regulatory immune mechanisms broadly enhance or activate the immune system. As a result, activation is supraphysiologic, which can lead to immune-related adverse events (irAEs). This paper reviews immune checkpoint inhibitors (ICIs) including their mechanisms of action, usage, associated irAEs, and their management.

## 2. Overview of Mechanisms of Action

ICIs induce anti-tumor immune responses by blocking immune checkpoints. These immune checkpoints play an important role in normal physiology to downregulate T cell responses, such as in autoimmune disease regulation. Two important immune checkpoint signaling pathways include the cytotoxic T-lymphocyte antigen 4 (CTLA-4) and programmed death 1 (PD-1) pathways.

**Figure 1:**
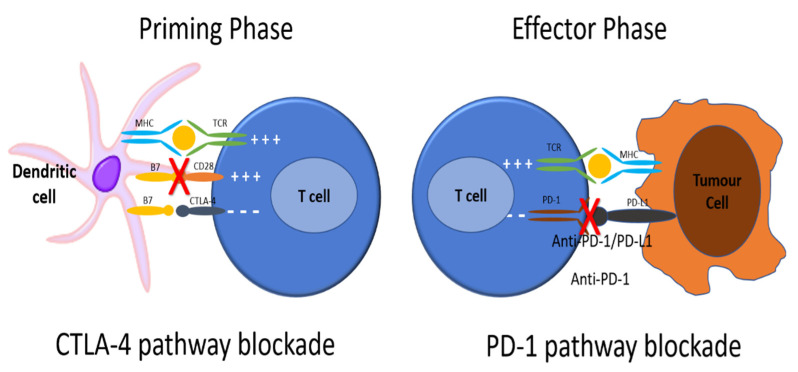
To be activated, T cells require major histocompatibility complex (MHC) class II molecules on an antigen presenting cell (APC) to present an antigen (Ag) that is recognized by the T cell receptor. Next, the CD28 receptor on the T cell is bound by CD80/86 on the APC, signaling the T cell to be activated. CTLA4 is found on T cell surfaces and competes with CD28 for binding to CD80/86 on the APC. When this interaction predominates, T cell activation signaling is attenuated. As well, PD-1 receptors are expressed on the surface of T cells. When PD-1 receptors are engaged by programmed death ligand 1 (PD-L1) on an APC, the T cell that recognizes the Ag being presented by the APC activates signaling pathways that downregulate activation and promote apoptosis. There is also reduced apoptosis of T-regulatory cells (Treg), facilitating downregulation of the immune response to that antigen. Cancer cells exploit these important physiologic mechanisms by upregulating PD-L1 expression on their cell surface, thereby attenuating any anti-tumor immune response through inducing the quiescence of tumor-reactive T cells. ICIs such as anti-CTLA-4, anti-PD-1, and anti-PD-L1 antibodies have been developed to restore the immune system’s ability to mount an anti-tumor immune response. Anti-CTLA-4 antibodies block the interaction between CTLA-4 and CD80/86 on the APCs, allowing for increased T cell activation. Anti-PD-1 and anti-PD-L1 antibodies block the interaction between PD-1 and PD-L1, allowing for increased T cell activation to Ag being presented by the tumor cell. In this way, the tumor cell is no longer recognized as “self”, tumor-reactive T cells are no longer shunted towards quiescence, and an anti-tumor immune response can be mounted.

To be activated, T cells require major histocompatibility complex (MHC) class II molecules on an antigen presenting cell (APC) to present an antigen (Ag) that is recognized by the T cell receptor, as well as engagement with other co-stimulatory molecules (Figure 1) [2,3]. The engagement of CTLA-4 and PD-1 receptors on T cells downregulates T cell activation. Cancer cells exploit these important physiologic immune checkpoints by engaging these receptors and attenuating a T cell-mediated anti-tumor immune response [2,3,4]. ICIs such as anti-CTLA-4, anti-PD-1, and anti-PD-Ligand 1 (PD-L1) antibodies have been developed to restore the immune system’s ability to mount an anti-tumor immune response.

## 3. Immune-Related Adverse Events

ICIs are used in a variety of disease sites as they universally upregulate the immune response, independent of the tumor-antigen being presented. In lung cancer, anti-PD-1 antibodies cemiplimab, nivolumab, and pembrolizumab, and anti-PD-L1 antibodies atezolizumab and durvalumab are used in the curative or metastatic settings to produce an anti-tumor immune response. However, the challenge with ICIs are the effects of a highly activated immune system on normal/non-tumor tissues via an autoimmune process, also called irAEs. irAEs are graded by severity (mild, moderate, severe, life-threatening, or death) on a scale from 1–5, respectively. In a meta-analysis of 36 phase II/III trials, the estimated incidence of any grade irAEs ranged from 54% to 76%, while the incidence of grades 3 and 4 adverse events ranged from 14.1% to 28.6% [5]. Interestingly, patients treated with ICIs and who develop an irAE have been shown to have a statistically significant reduced risk of death (~51%) and progression (~49%), suggesting that this may be proportional to the robustness of the T cell activation [6].

irAEs can occur in any tissue/organ system. Median onset ranges from 2–16 weeks from the start of treatment and varies depending on the organ system involved [2]. However, irAEs have been reported as early as one week after treatment commencement or as late as one year after discontinuation, presumably due to the presence of autoreactive T cell clones that remain in the body after treatment has stopped [7,8]. The most common irAEs are pruritis, rash, diarrhea, colitis, hypo- or hyper-thyroidism, and pneumonitis.

Combining one ICI with another ICI or conventional chemotherapy increases both the toxicity profile and severity in patients. Moreover, patients receiving the same ICI do not present with the same irAEs, even if being treated for the same type of cancer, suggesting there are patient/organ-specific microenvironments that can drive irAE [9,10]. While there are no known genetic risk factors for experiencing an irAE, personal risk factors such as a family or personal history of previous autoimmune disorder, high BMI, and elevated creatinine can increase the likelihood of ICI toxicity [11,12,13]. Interestingly, CTLA4 and PDCD1 polymorphisms are associated with autoimmune disorders [14,15].

## 4. How Do ICIs Cause Toxicity?

ICIs upregulate immune-activation pathways in a non-specific manner. Because of the differences in the types and frequencies of irAEs depending on the ICI used, there must be differences in the mechanisms by which they cause toxicity. As stated, normally CTLA4 maintains self-tolerance. Animal models and congenital genetic diseases in humans that cause functional abnormalities in CTLA-4 pathways demonstrate that abrogation of this regulation leads to T cell lymphoproliferation and lymphocytic infiltration, Treg defects, and auto-antibody (Ab) production [16,17,18]. In both mice and patients treated with CTLA-4 inhibitor, there are decreased circulating Treg and increased T helper (Th) 17 cells, whose enhancement is known to be involved in the pathogenesis of autoimmune diseases [19,20,21]. With respect to PD-1/PD-L1 inhibition, there are fewer circulating Treg cells in melanoma patients treated with ICI, and mice deficient for PD-1/PD-L1 develop auto-antibodies to various normal endogenous murine proteins [22,23,24,25,26].

T cell activation can also increase cross-talk between T and B cells, leading to increased auto-antibody production. In one study, patients treated with ICIs were shown to have changes in proportions of B cell populations, including reduced circulating B cells and increased CD21^low^ B cells and plasmablasts, which were highly indicative of subsequent irAEs [27].

Finally, ICIs may cause toxicity through cross-reactivity. When the immune system recognizes an Ag presented by a tumor cell and mounts a response, there may be cross-reactivity with similar Ag on normal/non-cancerous cells [28]. This is evidenced by the high number of melanoma patients with vitiligo after being treated with ICIs, and has also been demonstrated in fatal cases of irAE myocarditis at autopsy [7,29,30,31].

## 5. Organ-Specific ICIs

For patients on ICI, careful self-assessment by the patient and history gathering by the medical team is integral to identifying complications early. In this way, an attempt can be made to institute management strategies to both minimize the need to interrupt or discontinue ICI treatment, and prevent an irAE from progressing to higher grades with increased toxicity and morbidity for the patient. Using Cancer Care Ontario (CCO) and the American Society of Clinical Oncology (ASCO) guidelines, this section will briefly describe some of the more common organ-specific irAEs and their management [32,33]. A high yield summary of these sections can be found in Table 1, including definitions for how these irAEs are graded (Table 2). For any reference to steroid use, please see Table 1 for dosage recommendations.

### 5.1. Dermatologic

Cutaneous irAEs are most common, occurring in >30% of patients on ICIs, and tend to occur earlier than other organ-specific irAEs [34,35]. Generally, these occur irrespective of cancer type or number of treatments received, and more frequently with CTLA-4 inhibitors (~45%) [35,36,37]. Most are grade 1 or 2 toxicities; however, 1–3% of cases are grade 3 or higher, irrespective of the ICI [35]. Cutaneous reactions include lichenoid, psoriaform, granulomatous, eczematous and immunobullous reactions, vitiligo, drug rash with eosinophila and systemic symptoms (DRESS), toxic epidermal necrolysis (TEN), Stevens–Johnson syndrome (SJS), and Sweet syndrome [34,35]. More severe reactions include mucosal and palmoplantar surfaces. Interestingly, improved progression free survival (PFS) and overall survival (OS) have been reported in patients who develop cutaneous irAEs [38,39,40].

Per CCO guidelines, cutaneous irAEs are graded as 1–4 based on the percent of body surface area (BSA) covered, and type of reaction (Table 2) [32]. Any signs of desquamation should be considered a medical emergency and classified as grade 4. For grades 1 and 2 dermatitis, supportive therapy such as thick emollients is often all that is required. Topical steroids can be considered and are generally prescribed for grade 2 dermatitis, with anti-histamines to address pruritus as needed. These patients can be monitored while continuing on ICI. If the dermatitis persists, oral steroids and referral to a dermatologist can be considered.

If the patient has grade 3 or 4 dermatitis, a dermatologist consult is required. For grade 3 dermatitis, oral prednisone with a taper, and possible antibiotics are required. ICI should be held until resolution to grade 0 or 1, with the potential for re-challenge. If there is no improvement, or if the patient has grade 4 dermatitis (<5% of patients), the ICI should be permanently discontinued. Grade 4 dermatitis requires longer term IV steroids with a slower taper, as well as hospital admission for supportive care.

### 5.2. Endocrine

The incidence of endocrine irAEs ranges in the literature; however, one meta-analysis reported an overall incidence of clinically significant endocrinopathies of approximately 10% [41]. The most common endocrinopathies include acute hypophysitis and thyroid disease, though others such as development of type one diabetes mellitus (DM1), primary adrenal insufficiency (AI), hypercalcemia, and hypoparathyroidism occur more rarely [41,42]. Hypophysitis occurs most frequently with dual ICI or anti-CTLA4 Ab, whereas hypo- or hyperthyroidism occurs more frequently with PD-1 inhibitors [42,43]. Diagnosis of endocrine irAEs can be difficult due to their presentation with vague symptoms that can mimic a patient’s cancer such as fatigue, anorexia, and nausea. With the exception of labs assessing thyroid function and electrolytes including calcium, there are no routine laboratory tests that are monitored to detect endocrine dysfunction. However, some physicians using CTLA-4 inhibitors will assess other endocrine hormone levels (e.g., ACTH, testosterone, LH/FSH) at the onset of treatment to record the patient’s baseline for future comparison.

Endocrine irAEs are rarely grade 3–4, and though steroids can be given, efficacy is not well-established in improving the pituitary–thyroid–adrenal axis [44]. Generally, hormone replacement is sufficient, though in rare severe cases admission to hospital for supportive care and/or other investigations may be required. For both hypo- and hyperthyroidism, grade 1 adverse events (AEs) occur in an asymptomatic patient with altered thyroid stimulating hormone (TSH) levels. These patients can be followed and their thyroid chemistry monitored without stopping the ICI. In the case of grade 2 AEs, the patient may have moderate symptoms that necessitate treatment. This includes levothyroxine in the case of hypothyroidism. For hyperthyroidism, beta blockers are the mainstay of symptomatic treatment as well as hydration and anti-diarrheals. Agents such as methimazole or propylthiouracil in the case of hyperthyroidism may be less effective in ICI induced hyperthyroidism unless true Graves’ disease is present, and consultation with an endocrinologist is warranted in grade 3 or 4 cases [45]. Immune-related thyrotoxicosis generally transitions to hypothyroidism and ultimately requires thyroid hormone supplementation [46,47,48]. For any grade 3 or 4 hypo-/hyperthyroidism, hospitalization may be indicated for supportive management. Steroids have not been shown to decrease the duration of toxicity [49]. If grade 2 or 3, ICI should be withheld until the patient is stable on hormone therapy and prednisone has been tapered to <7.5 mg of prednisone equivalents per day, with the possibility of re-challenge. If grade 4, re-challenge can be reasonable once the patient is stable on hormone replacement, although individual patient situations need to be considered [33].

Hypophysitis typically presents as low TSH and low free T4. It occurs more frequently in males, and after approximately 2–6 months of treatment with an ICI [50]. Symptoms can be vague, or mimic the patient’s cancer, and so diagnosis can be delayed [51]. Once suspected however, endocrinology should be involved. Chemistry to assess morning cortisol, adrenocorticotropic hormone (ACTH), luteinizing hormone (LH), follicle stimulating hormone (FSH), and growth hormone (GH) will confirm the diagnosis. Imaging to rule out a cause that would require intervention should be completed. If a patient has grade 1 hypophysitis, monitor closely and continue the ICI. If the patient has grade 2 hypophysitis, withhold the ICI until grade 0–1, and re-challenge if the patient is stable on hormone replacement and asymptomatic. As with other endocrinopathies, if grade 3 or 4 hypophysitis is identified, the ICI may be resumed with appropriate hormone supplementation and monitoring once the initial clinical symptoms resolve.

Adrenal insufficiency (AI) as an irAE is rare though can be life-threatening. It can manifest as primary (0.7% and 4.2% of cases with single or double ICI, respectively [52,53]) or secondary AI (via hypophysitis), depending on the target of autoimmune antibodies. If primary AI, it is imperative to rule out inciting causes such as sepsis, which can present similarly, and complete a CT of the adrenals to assess for hemorrhage or metastases [54]. In general, once the patient reaches at least grade 2 AI, corticosteroids should be initiated, with likely long-term replacement required. In grades 2 and higher, ICI should be withheld until the irAE reaches grade 0 or 1, though re-challenge with ICI can resume once the patient is stable on hormone replacement and asymptomatic.

### 5.3. Gastrointestinal

Diarrhea is a common irAE occurring in ~35%, 20%, and >40% of patients on CTLA-4, PD-1 inhibitors, or combination therapy, respectively, though colitis is found in only 12%, 1%, and 14% of patients, respectively [55,56]. Similar symptoms occur with enteritis; however, constipation due to inflammation and abdominal pain are also possible presenting features. Typically, the work-up consists of a history and physical exam, as well as stool samples to rule out infection. Depending on the severity and type of symptoms elicited, computed tomography (CT) to evaluate for perforation or the extent of inflammation can be helpful. The median time to onset of diarrhea/colitis is 6–8 weeks for ipilimumab and nivolumab, and 3–4 months for pembrolizumab [57]. Therapy can range from supportive therapy with loperamide, in the case of grade 1 irAEs, the addition of IV hydration, electrolyte replacement, and oral steroids in the case of grade 2 irAE, to IV steroids and possible antibiotics with hospital admission for supportive care in the case of grade 3 or 4 irAEs. For grade 1 AEs, ICI can continue as long as patient symptoms are controlled with supportive therapy. For grade 2 AEs, ICI should be withheld until grade 0 and the patient is on <7.5 mg/day prednisone equivalents if on a CTLA-4 inhibitor, or <10 mg/day prednisone equivalents if on a PD-1 inhibitor. For grades 3 and 4 diarrhea, ICI should be discontinued. In some cases, the addition of infliximab is necessary, though caution is advised with grade 4 AE due to the risk of bowl perforation.

Liver toxicity can occur in 1–17% of patients on ICI [51,58,59]. The incidence varies by regimen with those on CTLA-4 inhibitors occurring slightly more commonly. Most events are grade 1 or 2. Liver chemistry should be reviewed with each ICI cycle as asymptomatic elevations in transaminases are the most common initial presentation [51]. There are many reasons a patient with cancer on ICI can present with hepatitis; therefore, alternative diagnoses should be carefully considered and can include disease progression, thrombosis, drug-induced liver injury, acute infections, alcohol-induced hepatitis, effects of other concomitant systemic therapies, as well as irAEs. If grade 1, the patient can be monitored while continuing on ICI. If grade 2, prednisone should be initiated with a slow taper provided liver transaminases normalize with treatment. Once normalized, and provided the patient is on ≤10 mg prednisone equivalents per day, re-challenge with ICI can be considered. If grade 3 or 4, hepatology/gastroenterology should be consulted, and biopsy considered. In such cases, high dose IV steroids are required with a long taper. If there is no downward trend to suggest the resolution of transaminitis by 3 days, mycofenolate mofetil (MMF) should be added. Again, if there is no improvement by 7 days, a switch to another immunosuppressant such as tacrolimus should be made. As a last resort, infliximab, after consultation with expert opinion and the patient, can be considered, as it may also cause transaminitis. If the hepatitis is grade 3 or 4, the ICI should be permanently discontinued.

### 5.4. Lung

Though pneumonitis as an irAE is rare (<5% of cases and <1% meeting criteria for grade 3 or 4 toxicity, or <10% of cases for mono- and combination therapy, respectively), it can be life-threatening and warrants careful consideration [60,61]. Patients can present with a dry, unproductive cough, tachypneic, dyspneic, tachycardic, cyanosed, and/or fatigue [61]. They can have exertional hypoxia, or hypoxia as lung inflammation and interstitial/alveolar infiltrates increase [61]. Grade 1 disease is asymptomatic and requires no intervention; however, prednisone can be considered and oxygen saturation, chest x-ray (CXR), or possibly CT should be completed with subsequent cycles. If the patient is placed on steroids for any reason, consider withholding the ICI until resolution. Patients are symptomatic with grade 2 irAEs and therefore require medical intervention. Respirology and infectious disease consults are recommended, as well as starting prednisone with a long taper once resolution begins. If there is no improvement by 48–72 h, the patient should be approached as grade 3 or 4 pneumonitis and the ICI should be withheld until symptom resolution and the patient is on <10 mg/day prednisone equivalents. Re-challenge can occur, but if toxicity recurs, the ICI should be discontinued.

Grade 3 and 4 pneumonitis are treated the same. Respirology and infectious disease consults are recommended, as well as consideration for bronchoscopy/biopsy to aid with the diagnosis. High dose steroids should be initiated with a long taper once symptoms improve. If there is no improvement at 48 h, additional immunosuppression with infliximab should be added to the treatment. Supportive care such as oxygen and prophylactic antibiotics should be included; the ICI should be permanently discontinued.

### 5.5. Renal

Nephritis is typically asymptomatic at onset and is found with rising serum creatinine on routine labs [62]. On progression, symptoms can include edema, oliguria, and other electrolyte abnormalities. Renal toxicity occurs in <5% of patients [63,64]. The management of grade 1 disease includes hydration, cessation of nephrotoxic medications, and correcting electrolyte imbalances. ICI can be continued provided creatinine stabilizes or decreases. Grade 2, 3, and 4 nephritis are treated similarly. As with other irAEs, other causes of elevated creatinine should be ruled out with urine microscopy and ultrasound +/− biopsy; nephrologist consultation should be considered. For grade 2 AE, prednisone should be started and tapered once creatinine reaches grade 0 or 1 levels. The ICI should be withheld until creatinine decreases to grade 1 criteria, and the patient is on <10 mg/day of prednisone equivalents. For grades 3 and 4 AE, methylprednisolone should be initiated with a slow taper once resolution occurs. Addition of MMF could be considered in refractory cases, and hemodialysis may be necessary. If grade 3 or 4 irAEs, discontinue the ICI.

### 5.6. Neurologic

There are a wide variety of neurotoxicities at various degrees of severity that can occur due to ICI therapy, but occur in <5% of patients [65,66]. These include potential antibody-mediated toxicities such as paresthesias, Guillian–Barre syndrome, and myasthenia gravis, or other sensory, motor, and CNS toxicities like enteric neuropathy, inflammatory myopathy, lymphocytic meningitis, cerebral vasculitis, and optic neuritis [65,66]. Neurologic irAEs usually occur within one to six weeks of starting ICI treatment [65,66]. If grade 1, the ICI can be continued, and the patient monitored closely for progression. If grade 2 or greater, a neurology consult is recommended as well as magnetic resonance imaging, lumbar puncture, nerve conduction studies, or electromyography based on the patient’s symptoms to rule out other non-ICI-related causes. For grade 2, oral steroids should be initiated while ICI is withheld. Re-challenge can be considered once symptoms are grade 0 or 1, and a review with a multidisciplinary team to weigh the risks and benefits occurs. If grades 3 or 4, the ICI should be permanently discontinued and higher doses of prednisone to control the AE are recommended. For any grade 2–4 irAEs, consider a separate immunosuppressive agent if there is no improvement on prednisone (e.g., infliximab or MMF). Some patients may require IV immunoglobulin (IVIG), plasmapheresis, or other supportive approaches.

### 5.7. Cardiac

The incidence of cardiac toxicities is <1% for both single and double agent ICI, and presents as a wide variety of toxicities including myocarditis, pericarditis, arrhythmias, cardiomyopathy, and impaired ventricular function [33,60]. Though infrequent, cardiotoxicity can be life-threatening. If suspected, a consultation with a cardiologist is recommended, as well as holding the ICI and instituting high dose corticosteroids. If required, escalation to other immunosuppressive agents can be performed. It is unclear whether re-challenge should be conducted.

## 6. Efficacy of Immunotherapy after Treatment with Corticosteroids for Any Reason

Corticosteroids affect and attenuate numerous points along a pro-inflammatory pathway. As such, it was thought that perhaps glucocorticoids may reduce the efficacy of ICI, and ultimately patient outcomes. Because of this, patients on systemic glucocorticoids have generally been excluded from clinical trials of ICIs, leaving it to retrospective analyses to address the use of steroids in patients on ICI and their outcomes, and whether there is a causal relationship, or merely an association if outcomes differ.

One such retrospective study included 640 patients treated for NSCLC with single agent ICI, 14% of which were on corticosteroids ≥ 10 mg prednisone-equivalents per day, and 75% of whom had steroids prescribed for cancer-related dyspnea, fatigue, or control of symptoms from brain metastases [67]. Upon taking anti-PD-1/PD-L1 ICIs, patients experienced significantly lower objective response rates (ORR) and reduced PFS and OS, even when multivariate analyses were completed to take into account other potentially confounding variables [67]. As well, the use of corticosteroids or other immune-modulating medications (e.g., infliximab) to treat irAEs were not subsequently associated with decreased efficacy [67]. The authors hypothesized that commencing corticosteroids before initiating ICIs could reduce efficacy, though not once the patient had already responded to ICI. Though interesting, these conclusions should be interpreted with caution as patients on steroids for cancer-related symptom management may, at baseline, have a lower performance status than the comparator group, which would also confer a poorer PFS and OS, confounding the final analysis.

A more recent systematic review and meta-analysis assessing OS and PFS outcomes in patients with NSCLC treated with ICI +/− corticosteroids for any reason included 5461 patients from 14 studies [68]. The authors found that despite the retrospective nature, low quality studies, and significant heterogeneity and publication bias, the use of corticosteroids for any reason while on ICI significantly reduced OS and PFS versus patients who did not take corticosteroids [68]. A subgroup analysis stratifying patients by reason for corticosteroid use showed worse OS if being used for supportive therapy versus those in which they were used to manage brain metastases, though PFS was no different [68].

Another meta-analysis included 15 studies with over 14,000 patients of any cancer type who had corticosteroid administration before and/or after initiation of ICI treatment [69]. Corticosteroid use significantly reduced PFS and OS in cancer patients treated with ICI. In a planned subgroup analysis, the reason for corticosteroid use impacted efficacy: those for whom steroids had been prescribed for cancer-related symptoms had a shorter PFS and OS versus those patients for whom corticosteroids were prescribed to address an irAE where there was no detrimental impact on OS [69].

Finally, one study prospectively followed 341 patients with hepatocellular carcinoma (HCC) treated with ICI therapy alone to assess the differences between PFS, OS, and ORR between patients receiving and not receiving corticosteroid therapy [70]. Overall, corticosteroid use did not predict for worse OS, PFS, or ORR in uni-/multivariate analyses. However, corticosteroids prescribed for cancer-related indications were predictive for significantly shorter PFS and were associated with refractoriness to ICI. The authors proposed that this was due to the fact that patients with symptomatic HCC have a poorer prognosis, rather than a causal relationship between corticosteroid use and outcomes [70].

Generally, these studies use a threshold of 10 mg of prednisone equivalents per day, which is slightly above physiologic levels. In practice, if corticosteroids are required, patients should be on the lowest possible dose while on ICI, though they are not excluded from ICI therapy if they exceed the studied threshold of 10 mg of prednisone equivalents per day. It is reassuring that studies have demonstrated no adverse effect on the efficacy of ICI in patients prescribed corticosteroids to manage irAEs.

## 7. Re-Challenge with ICI after Interruption Due to irAE

When an ICI is held due to an irAE, a decision needs to be made with respect to re-initiating/re-challenging a patient with the drug due to concerns that re-challenge will be associated with recurrence of the irAE, which is especially worrisome if the event was of a higher grade or serious. Unfortunately, understanding which factors to take into account when weighing the risks and benefits of ICI re-challenge are unknown. One study that attempted to address this question found that patients who responded to an ICI prior to holding due to an irAE may not benefit from re-challenge, while for those without an OR at the time ICI was held, re-challenge was associated with improved PFS and OS versus those who were not re-challenged [71]. Though interesting, this study was of a small sample size, and there are reasons other than an irAE that can lead to discontinuation of the ICI that appear not to have been accounted for (e.g., worse performance status).

One observational study aimed to identify the rate of recurrence of the same irAE that prompted ICI interruption and management upon ICI re-challenge, in an attempt to identify clinical features associated with recurrence [72]. In a population of 452 cases of irAEs where re-challenge occurred, 28.8% of patients experienced recurrence of the initial irAE, with those on CTLA-4 or combination therapy experiencing recurrence most often. Colitis, hepatitis, and pneumonitis were associated with higher recurrence rates compared to adrenal events, which were found to have lower recurrence rates in a multivariate analysis [72]. Recurrence of a different irAE after re-challenge was reported in only 4.4% of cases, with colitis being the most frequent [72]. Similar recurrence rates have been shown in other studies [71,73,74,75].

Several approaches to re-challenge post-holding an ICI due to an irAE exist, including: (i) no re-challenge, (ii) switching to another class of ICI, (iii) re-challenge with the same ICI regimen, and (iv) restarting ICI with prophylactic immunosuppressive therapy. As above, depending on which organ system was affected and provided the irAE was lower grade, re-challenge with the same ICI is reasonable, and does not necessarily require prophylactic immunosuppressive therapy. There is insufficient evidence to support re-challenge with another class of ICI as traditionally ICI have not been studied in sequence in clinical trials; however, there are data to support ceasing doublet ICI treatment and continuing on with one of the single agent ICIs from the doublet regimen. If the irAE was severe enough, re-challenge is not recommended.

## 8. Hyper-Progression

A relatively new and controversial side effect of ICI use is the concept of hyperprogressive disease (HPD). There appear to be emerging data that a subset of patients treated with ICI experience rapid progression of disease and receive no benefit from IO. A full discussion on HPD is beyond the scope of this review; however, please see the Canadian consensus guideline by Dr. S. Laurie and colleagues for a review of the topic [76].

## 9. Conclusions

ICIs have become the mainstay of therapy in many cancer types, including lung cancers. Through their relatively non-specific upregulation of the immune system, a variety of irAEs can occur, the frequency of which depends on the patient, the cancer type, and the specific ICI antibody. Careful assessment of patients for irAEs through history taking, physical exam, and routine laboratory assessments are key to identifying irAEs at early stages, when they can potentially be managed more easily and before progressing to higher grades or more serious effects. Generally, most patients are eligible for re-challenge, and the use of corticosteroids to address an irAE is not associated with more poor patient outcomes.

## Figures and Tables

**Table 1:** High yield management guidelines, including steroid doses, for common irAEs. This table is an abbreviated high yield summary of the CCO Immune Checkpoint Inhibitor Management Clinical Practice Guidelines and the ASCO Management of Immune-Related Adverse Events Clinical Practice Guidelines [32,33].

irAE	Management	Corticosteroid/Other Medication Dosages
Dermatitis	G1/2—supportive care (e.g., thick emollients); monitor and continue ICI G2—topical steroids; can continue ICI as long as symptoms are tolerable, else hold until resolution to G0/1 G3/4—dermatology consultation G3—oral prednisone with taper, possible abx; hold ICI until G0/1 sx and consider re-challenge G4—longer term IV steroids with slow taper, hospital admission, discontinue the ICI	G1—emollients (e.g., urea-based cream, oatmeal baths, cool compress) G2—Topical steroids (e.g., 1% hydrocortisone cream, 0.1% betamethasone cream; Anti-histamines (e.g., diphenhydramine, hydroxyzine, rupatadine) G3—prednisone 0.5–1 mg/kg/d until symptoms resolve to G0/1, then taper over 2–4 wks if 0.5 mg/kg/d OR over 4 wks if 1 mg/kg/d G4—methylprednisolone 1–2 mg/kg/d IV, then taper over ≥4 wks once resolved to G0/1
Hypothyroidism	G1—monitor TSH G2—monitor TSH and fT4; levothyroxine; hold ICI until stable on hormone replacement G3/4—steroids, hospitalization for supportive management G3—hold ICI until stable on hormone replacement AND steroids tapered to <7.5 mg/d prednisone equivalents G4—hold ICI until stable on hormone replacement AND steroids tapered to <7.5 mg/d prednisone equivalents; consider discontinuation	G2—levothyroxine 0.5–1.5 mcg/kg if no heart disease or severe comorbidities; if severe heart disease or comorbidities levothyroxine 12–25 mcg daily and increase every 4–6 wks as indicated ** if pt has hypothyroidism AND adrenal insufficiency then start steroid 2–3 d before starting levothyroxine G3/4—methylprednisolone 1–2 mg/kg/d IV until G0/1 sx or patient baseline and taper over at least 4 wks
Hyperthyroidism	G1—monitor TSH G2—monitor TSH and fT4; beta blocker and hydration for symptom management G3/4—possible hospitalization for supportive management +/− methimazole/PTU for diagnosed Grave’s disease G3—hold ICI until stable on hormone replacement G4—hold ICI until stable on hormone replacement	G2/3/4—propranolol 10–40 mg QID/atenolol 25–50 mg/d; if Graves’ disease—methimazole (20–30 mg/d reduced after 4–6 wks to maintenance dose of 5–15 mg/d)/PTU (200–300 mg/d reduced to maintenance dose of 50–150 mg/d ** if pt becomes hypothyroid, initiate thyroid replacement as with hypothyroidism
Hypophysitis	G1—monitor, supportive therapy if sx G2—supportive therapy if sx, add steroid, withhold ICI until G0/1 and re-challenge once stable on hormone replacement and asx G3/4—mgmt. as with G2, and consider discontinuing the ICI if irAE was severe/life-threatening	G1/2—if AM cortisol <250 nM or random cortisol <150 nM, then hydrocortisone TID (e.g., 20 mg QAM, 10 mg QPM + QHS); consider thyroid hormone replacement if falling TSH +/− low fT4 and ** always replace cortisol for ~1 wk prior to initiating thyroxine G3/4—if residual toxicity (≤ G2) and pt on <10 mg prednisone/d, then consider restarting ICI
Adrenal Insufficiency	G1/2—consult endocrinology; monitor labs (e.g., cortisol, ACTH, aldosterone, renin) and determine if primary or secondary based on ACTH; if G1 continue ICI G2/3—initiate hormone replacement if needed and start corticosteroid; hold ICI until G0/1 sx and stable on hormone replacement after tapering G3/4—treat as G1/2 AND hospitalization, IV corticosteroids after ruling out sepsis; hold ICI until G0/1 sx and stable on hormone replacement after tapering G4—2–3 L of isotonic saline or 5% dextrose in isotonic saline immediately ** recommend a medic alert bracelet	G2—prednisone 60–80 mg PO daily tapering over 1 mos G3/4—IV stress dose corticosteroids (4 mg dexamethasone Q12H if dx unclear, or 100 mg hydrocortisone IV ×1 then 50 mg IV Q6H if primary AI) and taper to maintenance doses over 2 weeks upon discharge ** if primary AI consider whether mineralocorticoid replacement (e.g., fludrocortisone) is needed
Diarrhea/Colitis	G1—supportive (e.g., loperamide); consider steroids if no improvement after 24 h G2—supportive (e.g., loperamide, IV hydration, electrolyte optimization), steroids; hold ICI until G0 and pt is on <7.5 mg/d prednisone equivalents on anti-CTLA-4, or <10 mg/d prednisone equivalents on anti-PD-1; if no improvement in 72 h, treat as G3/4 G3/4—higher dose steroids +/− abx, and hospital admission for supportive care; discontinue ICI; consider infliximab	G1/2—Loperamide (2 tabs at onset of diarrhea with 1 tab at each subsequent episode, no more than 10 tabs/day; discontinue when diarrhea stops); prednisone 0.5–1 mg/kg/d until G0/1 then taper over 2–4 wks if 0.5 mg/kg/d OR over 4 wks if 1 mg/kg/d G3/4—methylprednisolone 1–2 mg/kg/d IV until improvement then slow taper over ≥4 wks; if no response after 3 d, start infliximab 5 mg/kg IV Q2 wks (caution with G4 due to perforation risk)
Hepatitis	G1—monitor G2—prednisone until transaminases normalize, with slow taper and re-challenge once on ≤10 mg/d prednisone equivalents; increase to higher dose if no response G3/4—consult specialist; consider biopsy; initiate high dose IV steroids with long taper; if no response by 3 days, initiate MMF, and if no response by 7 days, initiate another immunosuppressant	G2—prednisone 0.5–1 mg/kg/d until transaminases normalize, taper over 2–4 wks if at 0.5 mg/kg/d, OR over 4 wks if at 1 mg/kg/d G3/4—methylprednisolone 1–2 mg/kg/d until transaminases normalize, then taper with prednisone at 1–2 mg/kg/d over ≥ 4 wks; MMF 500–1000 mg BID and discontinue once prednisone at 10 mg/d; add other immunosuppressant if no response ^¥^
Pneumonitis	G1—monitor, supportive, initiate SaO2 and CXR/CT with each cycle prior to proceeding; consider steroid G2—specialist consult (respirology, ID); prednisone with long taper once G0/1; if no improvement by 72 h treat as G3/4; hold ICI until G0/1 and pt on <10 mg/d prednisone equivalents; can re-challenge but if toxicity recurs discontinue ICI; empiric abx if any suspicion of infection G3/4—specialist consult (respirology, ID) and consider bx; prophylactic abx for opportunistic infections, high dose steroids with long taper once G0/1; if no improvement by 48 h add additional immunosuppression with infliximab; supportive care with O2 as indicated, permanently discontinue ICI	G2—prednisone (or IV equivalents) 1 mg/kg/d and taper over ≥4 wks G3/4—methylprednisolone 2–4 mg/kg/d IV and taper over ≥6 wks; infliximab 5 mg/kg IV Q2 wks (if contraindicated due to risk of perforation, sepsis, TB, NYHA 3/4 CHF) then consider MMF (500–1000 mg PO BID) or another immunosuppressive agent)
Nephritis	G1—monitor, supportive care, discontinue nephrotoxic medications, correct electrolyte imbalances; continue ICI G2/3/4—r/o other causes of elevated Cr with urine microscopy, U/S +/− bx, and consider specialist consultation; MMF in refractory cases, possible need for hemodialysis G2—prednisone with taper once G0/1; hold ICI until G0/1 and pt is on <10 mg/d prednisone equivalents; if Cr is increased for >7 d or sx worsen, then treat as G3/4 G3/4—methylprednisolone with long taper once G0/1; discontinue ICI	G2—prednisone 0.5–1 mg/kg/d and taper over 2–4 wks if 0.5 mg/kg/d OR over 4 wks if 1 mg/kg/d; if no response treat as G3/4 G3/4—methylprednisolone 1–2 mg/kg/d IV and taper over ≥4 wks once G0/1
Neurotoxicity	G1—monitor G2/3/4—specialist consultation, sx-directed investigations (MRI, LP, NCS, EMG); consider adjunct immunosuppressive agent if no improvement on prednisone (e.g., MMF, infliximab) and other supportive approaches (e.g., IVIG, plasmapheresis) G2—steroids, hold ICI, re-challenge when G0/1 and with multidisciplinary input G3/4—higher dose steroid, discontinue ICI	G2—prednisone 0.5–1 mg/kg/d and taper over 2–4 wks if 0.5 mg/kg/d OR over 4 wks if 1 mg/kg/d; if no response treat as G3/4 G3/4—prednisone 1–2 mg/kg/d IV and taper over ≥4 wks once resolution to G0/1; MMF 500 mg BID; infliximab 5 mg/kg
Cardiotoxicity	G1/2/3/4—hold ICI; admit the patient and start high dose corticosteroids and obtain cardiology consult for sx management appropriateness of re-challenge is unknown, though consider discontinuation at G2/3/4	G1/2/3/4—prednisone 1–2 mg/kg daily and switch to methylprednisolone 1 g daily if prednisone ineffective; if refractory also consider other immunosuppressive agents (e.g., MMF, infliximab, ATG) ** Infliximab is contraindicated in patients with mod–severe HF as it is associated with HF itself

^¥^ Ex. Tacrolimus. Abbreviations: abx, antibiotics; ACTH, adrenocorticotropic hormone; AI, adrenal insufficiency; ATG, antithymocyte globulin; BID, bis in die (twice daily); bx, biopsy; Cr, creatinine; CT, computed tomography; CXR, chest x-ray; d, day; dx, diagnosis; EMG, electromyography; fT4, free T4; G, grade; H, hour; HF, heart failure; IV, intravenous; kg, kilogram; LP, lumbar puncture; mg, milligram; MMF, mycophenolate mofetil; MRI, magnetic resonance imaging; NCS, nerve conduction study; nM, nanomol/litre; NYHA, New York Heart Association; PO, per os; pt, patient; PTU, propylthiouracil; Q, quaque (each); r/o, rule out; SaO2, oxygen saturation; sx, symptoms; TB, tuberculosis; TSH, thyroid stimulating hormone; U/S, ultrasound; wks, weeks.

**Table 2:** Definition of grades of severity for various common irAEs. These definitions have been taken from the CCO Immune Checkpoint Inhibitor Management Clinical Practice Guidelines [32].

irAE	Grade 1	Grade 2	Grade 3	Grade 4
Dermatitis	Macules/papules covering <10% BSA +/− associated symptoms (e.g., pruritis, burning, tightness)	Macules/papules covering 10–30% BSA +/− associated symptoms (e.g., pruritis, burning, tightness) AND limiting ADLs	Macules/papules covering >30% BSA +/− associated symptoms (e.g., pruritis, burning, tightness) AND limiting self-care ADLs AND local superinfection	Life-threatening; SJS or widespread mucosal ulcerations (complicated rash with full-thickness dermal ulceration or necrosis)
Hypothyroidism	Asymptomatic; fT4 normal AND TSH >10 mUI/L	Moderate sx (e.g., fatigue, constipation, weight gain, loss of appetite, dry skin, eyelid edema, puffy face, hair loss); Low fT4 +/− TSH >10 mUI/L	Severe sx (e.g., bradycardia, hypotension, pericardial effusion, depression, hypoventilation, stupor, lethargy); very low fT4 and very high TSH	Life-threatening; extremely low fT4 and extremely high TSH (myxedema coma)
Hyperthyroidism	Asymptomatic; fT4 normal AND TSH suppressed (<0.3 mUI/L)	Moderate sx (e.g., weight loss, increased appetite, anxiety and irritability, muscle weakness, menstrual irregularities, fatigue, tachycardia); fT4 high AND TSH suppressed (<0.1 mUI/L)	Severe sx (e.g., arrhythmia, tremor, sweating, insomnia, diarrhea); fT4 normal AND TSH suppressed (<0.1 mUI/L)	Life-threatening; fT4 high AND TSH suppressed (<0.1 mUI/L)
Hypophysitis	Asymptomatic or mild sx (e.g., fatigue, weakness); clinical or diagnostic observations only	Moderate sx (e.g., headache, hypotension); limits IALDs	Severe or medically significant sx but not life-threatening; limiting self-care ADLs	Life-threatening consequences or any visual disturbances; urgent intervention indicated
Adrenal Insufficiency	Asymptomatic or mild sx (e.g., fatigue); clinical or diagnostic observations only	Moderate sx requiring medical intervention	Severe sx requiring hospitalization	Life-threatening adrenal crisis requiring urgent intervention (e.g., severe hypotension or hypovolemic shock, acute abdominal pain, vomiting, fever)
Diarrhea/colitis	<4 stools/day above pt baseline	4–6 stools/day above pt baseline AND associated abdominal pain, mucus, or blood in the stool	≥7 stools/day above pt baseline AND incontinence or need for hospitalization for IV fluids ≥24 h	Life-threatening; grade 3 sx plus fever or peritoneal signs consistent with perforation or ileus
Hepatitis (these ranges may differ if the patient is receiving ICI for HCC)	AST/ALT up to 3× ULN or t-bili up to 1.5× ULN (or <2× baseline)	AST/ALT >3× ULN or t-bili >1.5–3× ULN (or >2× baseline)	AST/ALT >5–20× ULN or t-bili >3–10× ULN	AST/ALT >20× ULN or t-bili >10× ULN
Pneumonitis	Asymptomatic, diagnosis is radiographic	Sx, medical intervention is indicated as it limits IADLs	Severe sx that limit self-care ADLs; supplemental O2 is indicated	Life-threatening respiratory compromise; urgent intervention indicated
Nephritis	Serum Cr > ULN AND >1.5–2× pt baseline; 1+ proteinuria (<1 g/24 h)	Serum >2–3× pt baseline; 2+ proteinuria (<1.0–3.4 g/24 h)	Serum Cr >3× pt baseline; proteinuria >3.5 g/24 h	Life-threatening; serum Cr >6× ULN; dialysis indicated
Neurotoxicity	Asymptomatic or mildly sx	New onset moderate sx limiting IALDs	New onset severe sx (e.g., vision changes, weakness, sensory deficits); affecting self-care ADLs; not life-threatening	Life-threatening; urgent intervention indicated
Cardiotoxicity	Abnormal cardiac biomarkers or ECG	Abnormal screening tests with mild sx	Moderately abnormal testing or sx with mild activity	Life-threatening; moderate to severe decompensation, intervention required

Abbreviations: ADLs, activities of daily living; ALT, alanine aminotransferase; AST, aspartate aminotransferase; BSA, body surface area; ECG, electrocardiogram; fT4, free T4; g, grams; HCC, hepatocellular carcinoma; IADLs, instrumental activities of daily living; mUI/L, milli-international units per litre; pt, patient; SJS, Stevens–Johnson syndrome; sx, symptomatic; t-bili, total bilirubin; TSH, thyroid stimulating hormone; ULN, upper limit of normal.
